# Processes for Designing Innovative Biomedical Hardware to Use in Space and on Earth

**DOI:** 10.1109/OJEMB.2023.3270393

**Published:** 2023-04-26

**Authors:** Kimia Seyedmadani, Keith A. Tucker, Baraquiel Reyna, Yasemin M. Akay, Metin Akay, Jennifer A. Fogarty

**Affiliations:** National Aeronautics and Space AdministrationJohnson Space Center 43834 Houston TX 77058 USA; University of Houston 14743 Houston TX 77204 USA; National Aeronautics and Space AdministrationJohnson Space Center 43834 Houston TX 77058 USA; National Aeronautics and Space AdministrationJohnson Space Center, Human Research Program 43834 Houston TX USA; Department of Biomedical EngineeringUniversity of Houston 14743 Houston TX 77204 USA; Translational Research Institute for Space Health and the Center for Space MedicineBaylor College of Medicine 3989 Houston TX 77030 USA

**Keywords:** Diagnostic, guideline, medical device, regulations, spaceflight

## Abstract

The new era of space exploration is increasing the astronaut's number and diversity in low orbit and beyond. The influx of such a diverse crew population will also increase the need for medical technologies to ensure safe and productive missions. Such a need represents a unique opportunity to innovate and develop diagnostics and treatment tools to meet future needs. Historically, terrestrial regulatory oversight of biomedical design processes was considered separate from spaceflight regulatory processes because it did not address spaceflight constraints. These constraints challenge the creative development of unique solutions for use in space. Translation between healthcare innovation in spaceflight to healthcare on Earth and vice versa requires understanding the commonalities, unique needs and constraints. This manuscript provides a framework for comparing Earth-space design processes and a perspective on the best practices to improve healthcare equity and health outcomes.

## I. Introduction

The growth of commercial human spaceflight has enabled humans other than government astronauts to travel to low Earth orbit (LEO) and soon beyond. Currently, the majority of human spaceflight is still driven by government agencies. These agencies consider many factors, such as time, cost, and technical rationale for planning space missions. Keeping crewmembers alive, healthy, and performance-ready despite spaceflight stressors' effects is paramount to any mission. The most common paradigm of a mission is to meet critical mission objectives while protecting crewmembers' health. The National Aeronautics and Space Administration's (NASA) approach reduces system and mission risks to the lowest achievable level. This requires a system engineering approach inclusive of the human system that identifies, prevents, and mitigates known risks and transparently identifies and accepts residual risks. Mission-relevant medical systems contribute to successful human spaceflight missions by addressing medical and performance risks that cannot be eliminated prior to the mission and those that may result from the mission [1], [2], [3]. In addition to addressing medical risks, the inclusion of a crew health and performance system [4], [5], [6], [7] presents the opportunity to enable human research sponsored by the NASA Human Research Program (HRP), other government agencies and space programs, and commercial entities. The sixty-one years of human presence in space have opened many opportunities for health research; however, the accessibility to these opportunities has been limited due to cost and regulations. Commercial space activities have recently increased the number of opportunities for flying mission-relevant systems and enabling human research outside of governments. Examples are the SpaceX free flyer missions (since 2020), Inspiration 4 (2021), the upcoming Polaris Dawn (2023) and commercial flyers accessing the International Space Station during Space Adventures and Axiom missions (since 2001 and 2022, respectively). On the other hand, commercial entities may approach space agencies' risk assessment differently. Alternative approaches may lack valuable lessons learned from governmental human spaceflights, and possible consequences may threaten human health and mission success. Long-term growth in health research opportunities requires knowledge sharing and best practices in designing, testing, and using human-system-relevant hardware and processes. We aim to establish a framework of “Best Practices” based on approaches by well-established regulatory entities for space biomedical instrumentation innovation to support the growth of human spaceflight. Our objectives are the followings:

1) Provide background about current terrestrial and extraterrestrial design and regulatory processes.
2) Compare and contrast those processes to identify gaps.
3) Provide success stories and examples of translation of healthcare innovation between Earth and Space.

Our goal is to raise awareness across fields to bring integrated solutions to improve healthcare equity and health outcomes in space and on Earth.

## II. Materials and Methods

This section provides a brief history of biomedical development methods, standards and regulations, followed by a process introduction and translation to innovative solutions.

### A. Biomedical Instrumentation Regulation History

The Medical Device and Diagnostics Industry (MDDI) has matured under the regulatory purview of the Food and Drug Administration (FDA) since 1848, which addresses the quality of the care and diagnostic systems in the United States with a series of “Best Practices Guidelines” (BPG) [10]. Under Code of Federal Regulation (CFR) Title 21 ss 820 subchapter H medical devices, quality system regulation of FDA, three categories of medical devices were identified based on their level of risk and associated hazards: Class (I) low risk, represents more than 43% of medical hardware (for example, the band-aid); Class (II) more complex systems and higher risk, representing more than 47% of medical devices (for example ultrasound system); Class (III) hardware that sustains or supports life and failure may cause a loss of life, represents 10% of medical hardware (for example implantable cardiac valve) [11]. The recent technological growth in the electronic and software industries has impacted the MDDI hardware development cycle by decreasing the time of development, operation and setup of hardware; on average, it takes 5–7 years, depending on classification, for design input to manufacturing [12]. Other countries followed the United States' example regulatory pathway for ensuring the safety and efficacy of designed healthcare hardware; some of the major regulatory groups are as follows, Pharmaceuticals and Medical Devices Agency (PMDA; Japan), National Medical Device Directorate (Iran), Medical Devices and Medical Equipment CEmark (Conformitè Europëenne (CE) Mark, European Union). This worldwide regulatory approach to medical device development created an expectation of safety and quality of care shared across several countries.

### B. Aerospace Medicine Regulation History

The history of aerospace medical capabilities dates back to 1785 when physicians John Jeffries and John Blanchard carried survival equipment on a balloon ride. Initially, commercially available and physician-designed systems were used [8], [9]. Weightlessness, thermal, radiation, hoover, and isolation, among other stressors of spacecraft and space environments, prompted the development of life support, safety, and health solutions. Although aviation medicine has historically been used to ensure human spaceflight safety, more human spaceflight research is required to understand how humans can survive and perform in increasingly extreme missions. NASA and other agencies have continued the human and life science research legacy. Research pipelines have been developed based on recognized risks and gaps in knowledge to inform and de-risk future exploration missions.

In 1990, during the design phase of the International Space Station (ISS), many countries, including Russia, Japan, Canada, and many others from the European Union, signed a Memorandum of Understanding (MOU) and developed Article 11.4 for establishing Multilateral Medical Policy Boards, the Multilateral Space Medicine Board, and the Multilateral Medical Operations Panel. This MOU states the level of care standards for treatment and medical operations when a crew is in space. The development of medical devices and diagnostics for astronauts is geared towards keeping the crew alive, healthy, and performance-ready. Contributing space agencies are responsible for developing standards and requirements prospectively to maintain the crew's health and performance readiness. The providing agency is in charge of the hardware's development [13], which may also be used by visiting spaceflight participants sponsored by contributing space agencies [14], [15]. The healthcare systems on board ISS (including the Integrated Medical System, the Crew Health Care System, the Russian segment medical hardware and other medical hardware) result from a diverse and multicultural approach to healthcare to make it potentially available to any crewmember part of an ISS mission.

The NASA Human Systems Risk Board is a collaborative forum where the operational and research communities discuss and debate the evidence for risks and mitigation strategies for several design reference missions. Its objective is to prioritize risks and methods of mitigation. There are two classifications for spaceflight biomedical hardware based on its state of development and the risk to mission success. Biomedical hardware addresses a medical or healthcare need that must be validated in its intended use environment. Hardware development categories are Information Technology Systems, Custom Development, Commercially available off-the-shelf (COTS), or Modified Commercial Systems (MOTS) [4]. In addition, NASA's criticality classification adds a layer of complexity and validation to the hardware design processes. Criticalities are risks and consequences based on a lower number associated with a higher consequence, in this case, Loss of the Crew (LOC) or Loss of the Mission (LOM) [6], [16], [17]. Based on criticality, a series of work instructions are utilized for design. The design and development of hardware for space exploration do not tolerate gratuitous extras and must be conscious of mass, power, volume, time, reliability, and user capabilities. Consideration of these constraints provides an opportunity for innovative hardware development.

### C. A Systematic Approach

The processes guiding development for Earth and space differ based on acceptable risk and required precision. This paper focuses on FDA Center for Devices and Radiological Health hardware guidelines (Class I, II & II medical device and diagnostics). These were reviewed against the aerospace industry biomedical hardware design processes with a heavy focus on NASA processes. Commercial entities and other space agencies have similar design and hardware quality processes with minor differences key factors are similar and followed as they represent best practices. Evaluation of the detailed product design and development methodologies used by bioengineering and aerospace (standards, regulations, and statement of the work) allowed the identification of convergence points between the design philosophies presented in the Result section. A systematic review of the NASA database “Spin-off,” “Grant announcement,” and “NASA Technical Reports System (NTRS)” was performed to ascertain examples of cross-over technologies between Space and Earth with keywords of “spacecraft,” “biomedical,” “Medical,” “terrestrial,” and “diagnostic.” The inclusion criteria were devices currently used in the medical field. Although there have been pharmaceutical experiments during spaceflight, such as AMG 785/CDP7851 in space transport system flight - 135, all pharmacological and drug delivery studies were excluded due to design processes and regulation differences [18], [19], [20], [21].

## III. Results

### A. Earth Biomedical Hardware Design Processes

**Fig. 1.**
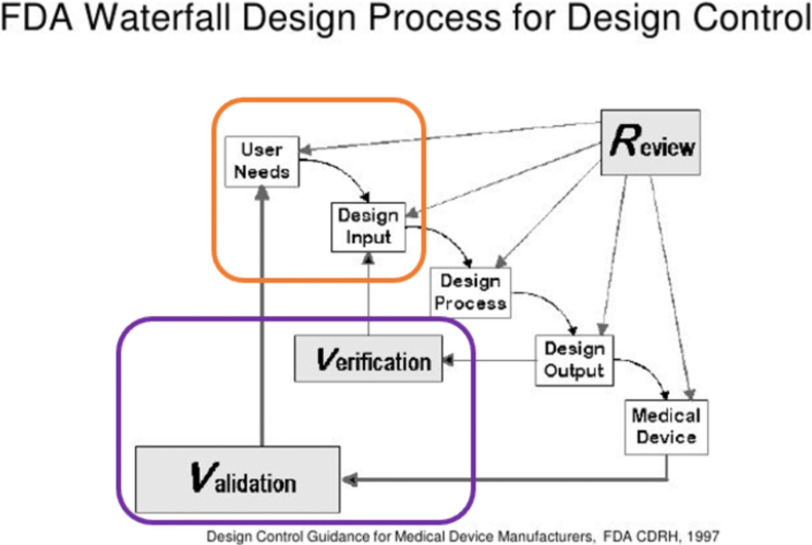
The FDA waterfall design published in FDA 21 CFR 820.30 and sub-clause 4.4 of ISO 9001 -1997 process highlighted in orange is the user input and the validation and verification process in purple. These are the two major areas that we found to be the most divergent between the MDDI and space biomedical hardware development processes.

Developing the right solution for an existing healthcare need requires significant effort and due diligence. Regardless of the FDA's classification, there is a recognized process to document and describe the medical or health need being addressed by the hardware and identify the user, which may be a trained healthcare provider or a patient or caregiver with no medical or technical knowledge. One of the design process methods followed in the medical device and diagnostic industry is the FDA waterfall, see Fig. 1, describing well-defined processes to provide a product that meets the customer's needs. Each phase has a review and regulation based on title 21 CFR 820 [22]. Below, we summarize critical aspects:

• **User Need:** defined as the identification of customer and stakeholder requirements and needs by conducting industry surveys, clinical observation, technical exchange, and deep dive into current solutions on the market, then translation to engineering matrices and developing a house of quality. House of Quality is a design tool that connects customer needs in methods and ways to achieve benchmarked performance requirements and define the final hardware specification (ISO13485- Table I).

**TABLE I:** List of Series of Regulations and Standards Used for Medical Hardware Development in Space and on Earth

**Label**	**Title**	**Standard Or Regulation**	**Earth Or Space**
NPD 7120.4	Engineering and Program/Project Management Policy	R	S
NPD 7120.4	Systems Engineering Processes and Requirements	R	S
NPR 7123.1	Experimental Flight Hardware Class I-E Procedural Requirements	R	S
JPR 7120.10	JSC Software Engineering Policy	R	S
JPD 7150.1	Environmental Testing	S	S
STANAG 4370	Mechanical Environmental Tests	S	S
AECTP 400	Standard Practice for System Safety	S	S
MIL-STD-882	Hazard Assessment Tests for Non-Nuclear Munitions	S	S
MIL-STD-2105	Testing and Calibration Laboratories	S	S
ISO/IEC 17025	Medical Device Quality Management Systems.	S	E
ISO 13485	Medical Device Risk Management	S	E
ISO 14971,	Software Used in Medical Devices,	S	E
ISO 62304,	Biological Evaluation of Medical Devices.	S	E
ISO 10993,	Symbols to be Used with Medical Device Labels, Labeling,	S	E
ISO 15223,	Safety and essential performance of medical electrical equipment	S	E
IEC 60601	Design Control	R	E
21 CFR 820.30	Traceability	R	E
21 CFR 820.60	Acceptance	R	E
21 CFR 820.86	Labelling and Handling	R	E
• **Design Input:** After identifying specifications, design input is a continuous and iterative process that organizes and prioritizes the identified need and specification for design.
• **Design Process:** All inputs are collated into various possible solutions that go through a series of design reviews by stakeholders until a final solution is identified (ISO10993, 15223, IEC 60601 and IEC 17025 Table I).
• **Design Output:** A prototype solution goes through several rounds of testing to verify that the design meets the documented user needs per the intended use during product design review (PDR) 21CFR 820.30 Table I.
• **Design for manufacturing and sustaining:** Production of a device often requires quality checks at each manufacturing site. Sustaining and repair strategies for the hardware are developed depending on the intended use environment(s) 21CFR 820.86 and 21 CFR 820.120 Table I.
• **Verification and validation:** verification means confirmation requirements are met by examination of objective evidence and heavily relies on evaluating data from testing and validation on clinical observation [23]; validation is achieved by a review of objective evidence that device specifications conform with user needs and intended use.

### B. Space Biomedical Hardware Design Processes

**Fig. 2.**
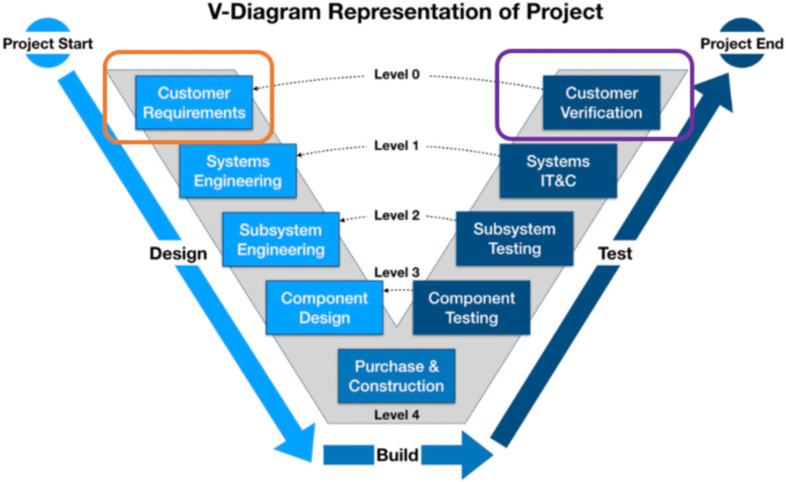
The V- diagram is the design paradigm in engineering focusing on a defined requirement for the final verification stage. In orange, customer requirements are highlighted, and customer verification in purple indicates differences between MDDI and spaceflight processes.

The traditional V- diagram design process is commonly used in the aerospace industry; see Fig. 2 [3], [5], [24], [25]. Below are a few details:

• **Customer requirements:** A series of requirements defined by the customer are provided to the design team.
• **System engineering phase:** a feasibility assessment of the flight hardware, project management plan, and possible concept of operations is developed, followed by identifying subsystems and finalizing the requirements list. Next is the identification of the requirements specification, which defines the requirements for the hardware and possible interface document with the vehicle and software developments resulting in a system requirement review NPD 7120.4, NPR7123.1 and JPR 7120.10 Table I.
• **Component design:** A Preliminary Design Review (PDR) is conducted at an early stage of the design and incorporates a review of all requirements, identifies design needs, drawings, data, software packages, verification plans, and safety, followed by test plans and training requirements. The prototyping starts after finalizing test methods and success and failure criteria for testing. The detail design phase is when a solution is defined, and risks, hazards and mitigation methods are identified for critical design review. At that time, all design plans are finalized, and the building starts in accordance with MIL-STD-882, AECTP 400, STANAG 4370 and MIL- STD-2105 Table I.
• **Purchasing and Construction:** Begins upon final approval of the design and integration plan. After proceeding through the design phases, the flight certification process begins with verification and is required for mission acceptance test performance. Government Certification Approval Request (GCAR) and System Acceptance Review (SAR) are NASA's final two steps of the verification process; note that the sustaining plans for hardware, if needed, are provided during the SAR. Note not all of the payloads require a GCAR; for example, a criticality level III payload for ISS does not require a GCAR process due to the low risk of impacting the mission or the crew [16], [26], [27].

## IV. Discussion

### A. Design Processes Similarity and Variance

Terrestrial and extraterrestrial divergence in hardware design starts from the first stage of the design process, user input, as shown in the orange in Figs. 1 and 2. General Electric (GE) Vivid Q Ultrasound, designed for terrestrial use, was adapted for spaceflight. However, due to the unique characteristics of human spaceflight, such as crewmembers not being trained medical personnel, the device had to be designed differently to accommodate this intended use. Crewmembers are given a maximum of two hours of training to familiarize themselves with the device before performing an ultrasound exam. The design input phase considered a special color-coded keyboard to help guide untrained operators during remote imaging procedures. This innovative approach for untrained operators may facilitate remote guidance of novice users during various conditions on Earth, such as those in remote and austere environments, socioeconomically deprived areas, and home healthcare, as demonstrated recently during the pandemic.

In addition to the user need for a variance, the intended use environment is no longer a hospital or medical office environment. As mentioned earlier, challenges arise from the limitations imposed by using in-flight resources and others from the space environment. Ultrasound images and data downlink with the limited bandwidth of ISS was a challenge. To overcome this, a video compiler system was designed to capture real-time ultrasound data, compile it into smaller packages, and send it autonomously to mission control for evaluation by a medical expert. This innovative approach was shared with the original manufacturer, who expanded its capability for use in limited resource areas on Earth.

Another point of difference between the two design processes is in Figs. 1 and 2. In Fig. 1, the verification and validation processes are well-defined and embedded in the biomedical design processes as part of the process, whereas they are separate from the V-Diagram Design phase and part of the Test phase; see Fig. 2. In MDDI, validation often happens after the hardware is fully developed and many potential subjects (‘n’) are available. This provides an opportunity to validate the requirements through clinical studies. In the case of space, the ‘n’ is often very small, less than 10, and in some studies, the ‘n’ can be as low as one subject [28]. Also, the validation stage is often a low priority and dismissed if verification has been achieved. This unique set of small crew allows for the development of custom-built or personalized healthcare solutions as the cost/benefit analysis balances the impact of the risk.

Finally, the sustaining plan on Earth versus space will vary greatly as the availability of resources, access to the supply chain, and hardware lifetime vastly differ between the two environments. However, developing a repeatable benchmark that is low profile to ensure the functionality of the hardware is crucial, as the diagnostic efficacy depends on the hardware's reliability in both Earth and space applications. In the case of ultrasound, the NASA sonographer developed a series of inflight images with a defined resolution; the crew-acquired image quality is compared to the engineering quality units on Earth every six months in addition to other functional hardware tests. This re-verification approach may provide us with a better understanding of true hardware lifetime under the extreme environment of space.

### B. Innovation From Space

Differences in the design processes and regulation of biomedical hardware development for spaceflight and Earth as influenced by the environment and operations (intended use) are an opportunity to innovate new technological solutions for healthcare. Table I contains standards and regulations used in spaceflight and military device development and FDA regulations that contribute to developing highly effective and safe healthcare devices. Examples of innovations from spaceflight-related research and operations to Earth are provided in Table II. Ultrasound II on the ISS was intended to be used for long-duration flight in extreme environments of spaceflight by minimally trained crewmembers as operators; the COTS system was modified in many ways, including but not limited to the electronic coating of circuit boards and hard drives as mitigation for radiation exposure and color coding of the keyboard for easier use. Accessibility to software and data from the ground and remote control was required for use in the spaceflight environment and was a modification requested by the manufacturer. This type of modification creates a capability that could be used for home healthcare and mobile healthcare units as well as in small clinics that are remotely located or serving populations in underserved and under-developed areas on Earth. Many other examples of such innovation are in the early stage or translated from space to Earth; see Table II. For example, in the 1980’s NASA was researching types of efficient lighting and wavelengths of light that are conducive to growing plants in extreme environments. Due to multidisciplinary collaborations, it was found that certain wavelengths of red-light [29] not only benefitted plant growth but also human health while isolated. This work led to healthcare applications on Earth and resulted in a spin-off of the Multi Radiance Medical Inc system for light therapy [30]. The unique challenges of human spaceflight provide opportunities to design for extreme environments that drive different approaches to healthcare solutions that may greatly benefit home healthcare and remote diagnostic and treatment capability. These technologies are not only multidisciplinary between healthcare and aerospace but are transdisciplinary, as demonstrated by the unique benefits of red light on agriculture and human health.

**TABLE II:** Innovative Spin-Offs List From Space to Earth

**Space Innovation**	**Technology Transfer**	**Year**
Remote Cardiac Monitor	NASA to Cardiology at Home	1965
Computerized Dynamic Posturography	Nasa Johnson Space Center to Zibrio	1970
Highly Accurate Position Detection	NASA Ames Center	1979
Light Therapy	NASA Marshall and Space Automation and Robotics	1980
Polymer for Prosthetics	NASA's Langley Research Center and MMA tech	1990
Telescope Mirror	Webb's mirrors into Johnson & Johnson Vision Care Inc	2000
“Weight”-Lifting Builds	Interim Resistive Exercise Device (IRED) to SpiraFle	2001
Monitors Patients at Home	Rachna Dhamija, CEO of Ejenta Inc	2008
Medical Imaging	NASA Life sciences to ImageIQ	2011
Remote monitoring	NASA to Velo's research	2012
Phototherapy treatments	NSBRI research NASA JSC to Harvard Medical School’	2013
Autonomous Medical Response Agent	TRISH and Nahlia (ongoing)	2018
Temperature Stable Basic Metabolic Panel	TRISH and Ativa Medical	2019
Active Pure Air filtration	Wisconsin Center for Space Automation and Robotics (WCSAR),	2020
Oculometric Cognition Testing	TRISH and Z3VR (ongoing)	2020

## V. Conclusion

We address the similarities and differences between biomedical design for terrestrial applications and extraterrestrial use, with examples of hardware applications that were translated between the two environments. However, there are many schools of thought on how to design hardware with respect to the intended use and acceptable risk. This document provides a perspective on the current best practices, resulting in solutions for space needs that have expanded capability and accessibility on Earth. We hope the perspective and insights provided increase innovation and the opportunity to utilize technologies from one sector to another to improve healthcare quality.
